# Adolescent breakfast skipping is associated with poorer academic performance: a school-based study from Hidhabu Abote District, Ethiopia

**DOI:** 10.1186/s41043-023-00424-z

**Published:** 2023-08-11

**Authors:** Dereje Feye, Tesfaye Gobena, Alexandra Brewis, Kedir Teji Roba

**Affiliations:** 1 College of Health and Medical Sciences, Haramaya University, Harar, Ethiopia; 2 School of Human Evolution and Social Change, Arizona State University, Tempe, USA

**Keywords:** Breakfast, Meal skipping, Adolescence, Ethiopia, Academic performance

## Abstract

**Background:**

Breakfast is regarded as “the most important meal of the day,” suggested to positively affect learning in children and adolescent in terms of cognitive and school performance. Yet, studies in LMIC settings are few and show very inconsistent results.

**Objective:**

To assess the prevalence and correlates of breakfast skipping and its association with school performance among randomly selected in-school adolescents in Hidhabu Abote Wereda, North Shewa Zone, Central Ethiopia.

**Methods:**

A cross-sectional study was conducted from November to December 2020. A total of 422 participants were selected randomly from high schools of Hidhabu Abote Wereda. Data were entered in to Epiata version 3.1 and exported to SPSS version 24 for analysis. Bivariate and multivariate binary logistic regression analysis identified factors that were significantly associated with the breakfast skipping. Odds ratio along with 95% Confidence interval was estimated to measure the strength of the association and level of statistical significance declared at *p*-value less than 0.05.

**Results:**

The magnitude of breakfast skipping was 41.3%, (95% CI (36.6–46.0)]. There was statistically significant association between breakfast skipping and overall academic performance [AOR: 5.18, 95% CI (1.54–7.46)], mathematics performance (3.88, 95% CI (1.34–11.22)], and English language performance scores [2.92, 95% CI (1.38–7.58)]. Being female [AOR = 1.857, 95% CI (1.05–3.27)], household food insecurity [AOR: 2.478, 95% CI (1.36–4.51)], and less maternal education [AOR 1.89, 95% CL (3.38–7.77)] were independently associated with breakfast skipping. The primary reasons given for breakfast skipping were lack of time, lack of appetite in morning, and concerns around weight gain.

**Conclusion:**

Nearly half of in-school adolescents were skipped breakfast meals, and reportedly in most cases for reasons unrelated to lack of food access. Students who skipped breakfast had lower levels of school performance.

## Introduction

Breakfast is regarded as “the most important meal of the day.” One assumption built into much nutritional programming globally is ensuring breakfast consumption (such as through school-based meal programs) will also improve school performance. In this study, we are interested in better recognizing and explaining connections between skipping breakfast and academic outcomes in the context of LMICs (lower and middle income countries), using a case of school children in a farming zone in Ethiopia.

Regular breakfast intake has been generally associated with better appetite, weight, and blood sugar control, fewer chronic disease markers, cognitive alertness, and academic performance in adults (e.g., [3, 32, 33] and academic performance in younger adults, i.e., university students Pengpid and Peltzer [31] noting the majority of such studies have been conducted in higher income countries. In LMIC where under nutrition is prevalent (as is the case in rural Eastern Ethiopia), knowing how and where interventions matter to improving both.

Yet, studies to date of how breakfast and school academic performance pattern together show inconsistent results overall; results are even less clear for LMIC countries in Africa and elsewhere, beyond that the provision of breakfast programs increases school attendance and inhibit dropping out, especially for the lowest wealth families (Drake et al. [15], Snilstveit et al. 2015, [9]. Across Africa, as other LMIC countries, there are relatively few studies with adolescents in particular documenting the relationship between breakfast and school performance, so the overall pattern is not established. For example, one study that considered school-provided breakfast for adolescents in South Africa found inconclusive results related to any apparent impact on school performance itself (noting that the school records were uneven) [21]. In Ikenne, Nigeria, a study of 190 secondary students found an association between eating breakfast and English and mathematics performance scores (Uwannah and Lotachi [42]). A study of 829 primary school students in Tehran, Iraq, found no correlation between breakfast skipping and academic performance (measured as overall performance assessments of “excellent” “good” or “needs more effort”) [38]. Similarly, in another of 14,473 in Kenya, also no association was found. In a study of 453 6–12-year-olds in Jordan, there was a clear correlation between parental reports of skipping breakfast and grade status [26]. A local study of middle school children in Mianyang City, China, found that more frequent eating of breakfast was associated with improved test-based comprehensive academic performance [13], a large-scale associational study of school records for 147,781 primary and middle schoolchildren in Jiangsu province similarly identified skipping breakfast was common, but also confirmed it was associated with worse academic scores [14]. In a study of 379 urban middle school children in India, those who regularly ate breakfast performed better in mathematics, English, and overall scores for the prior year (Gajre et al. [19]).

Most of these studies, as noted, have been conducted with primary and middle school children. But studies are arguably more necessary for adolescents, because—being in a transition to adulthood—they are more likely to be left to make breakfast decisions than younger children. And several studies reported that many adolescent students habitually skip meals, particularly breakfast (Boschloo, Ouwehand et al. [10]).

## Materials and methods

### Study area and period

The study was conducted from November to December 2020 in Hidhabu Abote district, Ethiopia. Hidhabu Abote Wereda is one of the 14 woredas of North Shewa Zone of Oromia Regional State and located 34kms from the zonal capital Fitche, and 146kms from the capital of Addis Ababa. The wereda has six high schools with 2575 total high school students contained in 20 kebeles (administrative units), 19 of them rural. The total population is 112,277 covering 485.84sqkms, with most household dependent for income on small-scale cash cropping, especially cereals (70.7). The most commonly spoken language is Afaan Oromo, and small population is Amharic speaker (Hidhabu Abote; Health office, Education office and Agricultural office, 2020).

### Study design and source population

A school-based cross-sectional study design was conducted among adolescents (14–19 years) attending high schools of Hidhabu Abote District.

### Sample size determination

Sample size was calculated using single population proportion formula by considering at 95% confidence level, 0.05 margin of error. We conservatively estimated average academic performance scores of 50% given that there were no other studies or pilot data from this particular study area. The required sample size was 422 after adding 10% of non-response rate.

### Sampling technique/procedure

Simple random sampling technique was used to select three schools from the six public high schools, with proportional allocation to each selected schools based on the number of students. Finally, participants were selected within each school using systematic random sampling technique. The K value is 3 for all, so after selecting the first sample by lottery method, every third student was selected as a study participant from the school’s roster of grade ten and grade nine students**.**

### Data collection procedures

#### Questionnaires

Structured questionnaires were given to adolescents for self-reporting. Family sociodemographic and economic status questions were adapted from EDHS [16]. Breakfast-eating practices were assessed by using FAO qualitative dietary diversity questionnaires and meal frequency questionnaires (FAO [43]). Following Khurshid, Mahmood et al. [27], breakfast was defined as a first meal of the day eaten before starting of daily activities before 10:00 am. Breakfast skipping was defined as reporting no breakfast was eaten on the day of data collection.

### Anthropometric measurements

Weight and height were directly measured, as important covariates. Weight was measured by a digital scale to the nearest 0.1 kg, without shoes and minimum clothes. Height was measured with portable stadiometer to the nearest 0.1 cm. The study participants were stand upright on bare feet, with heels together, and buttocks and back touching the meter rule. WHO 2007 growth charts reference were used to classify the BAZ (BMI for age). Z-scores were calculated using the Anthro Plus software.

### Data quality management

To maintain the quality and consistency of the data collection, 3-day training was given to data collectors. This covered the objective of the study, procedures, and ethical issues. A pre-test was conducted at Gebra-gurracha secondary school which is the nearby wereda in North Shewa Zone, and an additional one day of training was given with the final version of the questionnaire before the start of the actual data collection. All collected data were checked daily for completeness and consistencies by the supervisors and the principal investigators. To test for accuracy, the scales were checked by placing items of known weight on them after every 10 measurements. The scales was regularly checked and adjusted to zero after each measurement. To minimize measurement error, TEM was done before actual data collection with ten participants and take acceptable value for Intra-evaluator and Inter-evaluator less than 1.5% and 2%, respectively.

### Data processing and analysis

All completed questionnaires were checked again at data entry for completeness and consistency, and double data entry was done using the Epidata version 3.1 software. Then the data were exported to the SPSS version 24 for descriptive analysis and statistical modeling. Binary logistic regression analysis was used to test associations between variables; a p-value of less than 0.25 during bivariable analysis was entered into the final multivariate logistic regression model to control for identified confounders. Odds ratios along with a 95% confidence interval were estimated to measure the strength of the association. The level of statistical significance was declared at P-value less than or equal to 0.05. Absence of multi-collinearity was checked by calculating VIF, and model adequacy was checked by using the Hosmer and Lemeshow goodness of fit test.

### Ethical consideration

The study was approved by Haramaya University, College of Health and Medical Sciences Institutional Health Research Ethics Review Committee (IHRERC). Permission was obtained from Hidhabu Abote Wereda Education office and selected high schools before the study. Brief explanation about the objectives of the study was given for each selected student as a basis for assent. Written consent was obtained from the children’s family for those less than 18 years of age, and from students themselves if over 18 years old. Data collection took place during COVID-19, so both data collector and students wore masks and followed other basic public health protocols while measuring height and weight.

## Results

### Sociodemographic characteristics of the respondents

Sample descriptives are provided in Table 1. Out of 422 study participants invited, 402 agreed to participate in the study, making a response rate of 95.3%. 60.2% of the participant were from grade 9 and 39.8% were from grade 10. More than half, 57.2%, were females, and 91.8% of participants were in the age group 16–19 years. The median age of the study participant was 17 years with a minimum of 14 and maximum of 19 years.

**Table 1:** Socio-Demographic Characteristics of In-school Adolescents in Hidhabu Abote Wereda Oromia Region North Shewa, Central Ethiopia 2020

Variables		Frequency, *n* %
Grade	9	238 (59.2)
	10	164 (40.8)
Age group (in year)	10–15	33 (8.2)
	16–19	369 (91.8)
Sex	Female	230 (57.2)
	Male	172 (42.8)
Religion	Protestant	5 (1.2)
	Orthodox	377 (93.8)
	Muslim	14 (3.5)
	Waqeffana	6 (1.5)
Residence	Urban	102 (25.4)
	Rural	300 (74.6)
Number of family members	< 5	92 (22.9)
	> 5	310 (77.1)
With whom participant live currently	Alone	18 (4.5)
	Both	275 (68.4)
	Other than parents	109 (27.1)
Mothers Educational status	No formal education	238 (59.2)
	Primary	125 (31.1)
	Secondary	16 (4.0)
	College	11 (2.7)
	Degree and above	12 (3.0)
Fathers Educational status	No formal education	292 (72.6)
	Primary	91 (22.6)
	Higher level	19 (4.7)
Mothers occupation	House wife	357 (88.8)
	Government employer	21 (5.2)
	Merchant	24 (6.0)
Fathers occupation	Farmer	381 (94.4)
	Government employer	21 (5.2)
Average monthly income	< 2000ETB	104 (25.9)
	> 2000ETB	298 (74.1)
Time taken from home to school on foot	< 30 min	148 (36.8)
	> 30 min	254 (63.2)

### Prevalence of breakfast skipping

**Fig. 1:**
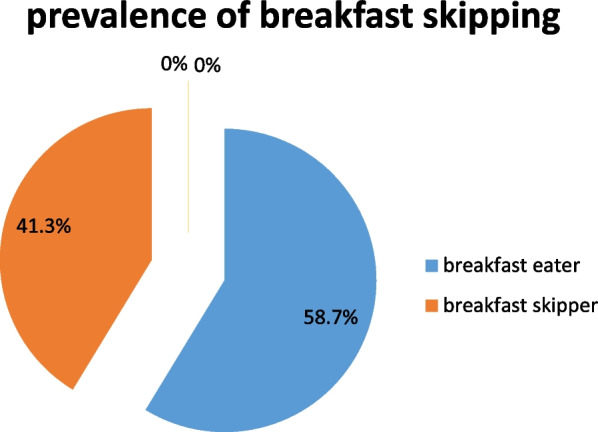
Prevalence of reported breakfast skipping among in-school Adolescents in Hidhabu Abote Wereda Oromia Region North Shewa, Central Ethiopia 2020

The prevalence of breakfast skipping among in this sample of school adolescents was 41.3% (Fig. 1).

### Breakfast practices

Of those who stated they had not eaten breakfast, the main reasons were (38.5%) identified it because they had no enough time (38.5%), another third said its (35.7%) poor appetite in the morning (35.7) and 13% due body image/shape concerns. Lack of available food was cited in only 1.5% of cases. For those who ate breakfast, one fourth (24.6) of them ate it on the way to school—noting that well more than half of surveyed students have a school commute over thirty minutes (Table 2).

**Table 2:** Breakfast habits reported by school adolescents’ in Hidhabu Abote Wereda Oromia Region North Shewa, Central Ethiopia 2020

Variable		Frequency	Percent
Eat breakfast today?	Yes	227	56.5
	No	175	43.5
Reason for breakfast skipping(for those who skip at least one day per week	No enough time	152	38.5
	Have nothing to eat	46	11.5
	No one prepare it for me	5	1.3
	Poor appetite in the morning	141	35.7
	For body image	51	13.0
Why you eat breakfast?	It makes me active	194	49.2
	Hungry in the morning	119	30.2
	Parents prepare it for me	46	11.7
	Parents will not motivate me if I not eat it	22	5.6
	It is one of the most important meal	13	3.3
Who prepare breakfast for you?	Mother	229	58
	My self	113	28.6
	Sister	18	4.5
	Others	35	8.9
Where do you usually eat your breakfast (only for regular eater)	From my home	178	75.4
	On the way to school	58	24.6
Any criteria to decide what to eat breakfast(for those who eat at least one day per week)	Food that is healthy	18	4.6
	Food that is cheap Food that is available in my home	43 331	11 84.4
Do you think there is anything your school could do to support or encourage you to eat healthy breakfast more often?	Yes	301	74.9
	No	101	25.1
How they support you if you say yes	School breakfast program	268	89
	Education on healthy breakfast	33	11

### Food consumption group eaten by participants during a week preceding the survey

Considering overall food consumption, based on what was eaten over the prior week, 98.8% of the participants were ate cereals every day, but 39.1% of the participants were never ate fruits and 69.4% had no meat (Table 3).

**Table 3:** Percentage of participants who consumed different food groups during a week preceding the survey in Hidhabu Abote Wereda (*n* = 402)

Food group	Never	1–2 days/wks	3–4 days/wks	5–6 days/wks	Every days
Cereals *n* (%)	0	0	0	5(1.2)	397(98.8)
Vegetables *n* (%)	24(6)	113(28.1)	23(5.7)	153(38.1)	89(22.1)
Fruits *n* (%)	157(39.1)	137(34.1)	69(17.2)	39(9.7)	0
Meat *n* (%)	279(69.4)	84(20.9)	37(9.2)	2(0.5)	0
Milk *n* (%)	215(53.5)	129(32.1)	57(14.2)	1(0.2)	0
Oils *n* (%)	0	0	26(6.5)	64(15.9)	312(77.6)
Pulses *n* (%)	1(0.2)	95(23.6)	251(62.4)	55(13.7)	0
Sugars *n* (%)	141(35.1)	1(0.2)	12(15.4)	167(41.5)	31 (7.7)

### Food consumption score of the respondents

**Fig. 2:**
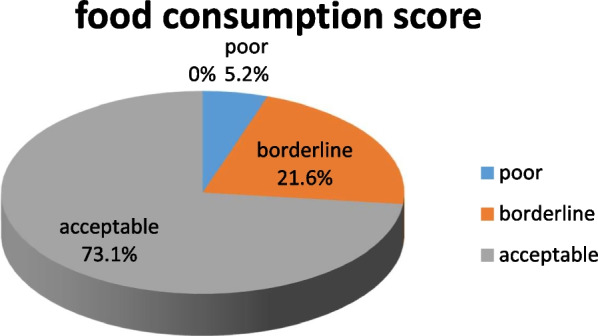
Food consumption score of school adolescents during a week preceding the survey in Hidhabu Abote Wereda Oromia Region North Shewa, Central Ethiopia 2020

We calculated a food consumption score based on reported frequency of eating eight food groups. Food consumption status is based on the following thresholds: 0–21: poor; 21.5–35: borderline; > 35: acceptable which is based on WFP food consumption score thresholds (WFP 2008). In our case, 73.1% had food consumption that could be classified as “acceptable,” 21.6% had “borderline,” and 5.2% “poor” (Fig. 2).

### Nutritional status and meal frequency characteristics of the respondents

Among the study participants, 14.9% had BAZ < − 2 (underweight) and 82.8% were in the clinically normal range. Among the respondents, 48% reported their actual daily meal pattern to be less than 3 times which is below the daily recommended meal frequency for adolescents. Among study participants, 74.6% said they did not ever eat snacks or between meals in the preceding week (Table 4).

**Table 4:** Food consumption score and meal frequency *distribution* of school Adolescents in Hidhabu Abote Wereda Oromia Region North Shewa, Central Ethiopia 2020

Variable		Frequency	Percent
How many meals you typically eat in one day	< 3 times	193	48
	> 3 times	209	52
How many times you typically eat between meals in one day	1 times	101	25.1
	2 times	1	0.2
	Never eat it	300	74.6
Nutritional status based on BAZ	Underweight	60	14.9
	Normal	333	82.4
	Overweight	9	2.2

### Relationship between breakfast skipping and school performance

To find the association between breakfast skipping and school performance, first we categorized students’ average overall academic scores into high performance (“A”) those who scored ≥ 80, medium performance (“B”) those scored 60–80, and low performance(“C”) those scored < 60 based on the standard scales of the Ethiopian Ministry of Education. There proved to be a statistically significant relationship between student's score and skipping breakfast. The majority of the students who skipped breakfast had grade "C" (low performance) (*p* = 0.008), while 87.5% of the students who did not skip breakfast had grade "A"(high performance). There were also statistically significant relationships between student’s math score and English language scores and breakfast skipping (*p* = 0.014 and 0.012, respectively) (Table 5).

**Table 5:** Multivariable logistic regression analysis of factors associated with breakfast skipping among in-school adolescents in Hidhabu Abote Wereda

Variable	Category	Breakfast status		COR (95%CI)	AOR (95%CI)
		Breakfast skipper *N*(%)	Breakfast eater *N*(%)		
Gender	Female	116 (50.4)	114 (49.6)	2.48 (1.63–3.77)	1.86 (1.05–3.27)*
	Not female	50 (29.1)	112 (70.9)	1	1
Household size	> 5 person < 5 person	138 (44.5)	172 (55.5)	1.83 (1.12–3.02)1	0.94 (0.43–2.03) 1
Residence	Urban	28 (30.4)	64 (69.6)	0.71 (0.45–1,14)	1.01 (0.47–2.18)
	Rural	36 (39.1)	66 (61.9)	1	1
Mother education level	No formal education	125 (52.5)	113 (47.5)	7.55 (2.84–19.90)	1.89 (3.38–7.77)*
	Primary	36 (28.8)	89 (71.2)	2.75 (0.99–7.59)	4.33 (0.91–20.60)
	Higher level	5 (12.8)	34 (87.2)	1	1
Monthly income of the family	High income	78 (26.2)	220 (73.8)	0.06 (0.04–0.12)	0.07 (0.04–0.14)*
	Low income	88 (84.6)	16 (15.4)	1	1
Time taken from home to school on foot	> 30 min	113 (44.5)	141 (55.5)	1.44 (0.95–2.18)	1.42 (0.79–2.55)
	< 30 min	53 (35.8)	95 (64.2)	1	1
Food security status	Food insecure	77 (65.8)	40 (36.4)	4.24 (2.69–6.69)	2.48 (1.36–4.50)*
	Food secure	89 (31.2)	196 (68.8)	1	1
Total average score	Low performance (C)	92 (57.9)	67 (42.1)	9.61 (3.58–25.89)	5.19 (1.54–7.46)*
	Medium performance (B)	69 (34)	134 (66)	3.60 (1.35–9.61)	2.00 (0.60–6.66)
	High performance (A)	5 (12.5)	35 (87.5)	1	1
Math’s score	Low performance (C)	88 (57.9)	64 (42.1)	7.857 (3.31–18.66)	3.89 (1.34–11.22)*
	Medium performance (B)	71 (35)	132 (65)	3.07 (1.31–7.21)	1.83 (0.7–5.17)
	High performance (A)	7 (14.9)	40 (85.1)	1	1
English score	Low performance (C)	100 (54.1)	85 (45.9)	11.18 (3.83–32.59)	4.93 (1.38–7.58)*
	Medium performance (B)	62 (35.4)	113 (64.6)	5.21 (1.78–15.29)	2.92 (0.83–10.34)
	High performance (A	4 (9.5)	38 (90.5)	1	1
Absent from the school at least one day	Yes	109 (56.5)	84 (43.5)	3.46 (2.28–5.25)	2.22 (1.25–3.96)*
	No	57 (27.3)	152 (72.7)	1	1

* Significant at *P*-value < 0.05, *COR* Crude odd ratio, *AOR* Adjusted odd ratio, *CI* Confidence interval

In multivariable logistic regression analyses, we were able to take likely covariates into account. Female participants were 1.857 times more likely to skip breakfast than male [AOR = 1.857, 95% CI (1.054–3.272)]. Participants with the average monthly family income of ≥ 2000 ETB were 7.1 times more likely to eat breakfast than those with an income < 2000ETB [AOR 0.071, 95% CI (0.036–0.139)]. Those in food insecure households were 2.477 times more likely to skip breakfast than those in food secure households [AOR 2.477, 95% CI (1.361–4.508)]. Participants whose mothers had no formal education were 1.984 times more likely to skip breakfast than others [AOR 1.894, 95%CL (3.379–7.772)] (Table 5).

## Discussion

Identifying how practices impact adolescent academics has implications for the design of intervention programming to improve both. Studies in LMIC are few, especially for adolescents. The current study revealed a high rate of breakfast skipping in this sample of Ethiopian adolescent schoolchildren (41.3%), although it is consistent with findings of a study done in Sidama Zone, Southern Ethiopia 42.3% [6]. It also falls within the range of prior studies conducted in other LMIC, such as Pakistan 47.3% [8], India 47.7% (Aarthi et al. [1]), Jordan 18.5% [5], and Nigeria 23% [12].These are all lower than some studies conducted in advanced economies, where rates can be extremely high—e.g., Korea 72.1%(Kim et.al [28]).

Unlike many of the few prior studies conducted in African schools samples, we found a positive association between breakfast skipping and lower academic performance, and this is evident even once such factors as household food insecurity and income were taken into account. Female students showed a stronger association. This finding is, however, in line with studies in other LMIC, such as those from Jordan, India, and China discussed above.

As could be expected, breakfast skipping was associated with lower family income and more food insecurity. But, importantly, the reasons provided for why meals were skipped were only rarely (11%) noted to be because of a lack of food access. Regarding reasons for skipping breakfast meal, it was noticed that more than one thirds (38.5%) of the students skipped breakfast because they had no enough time, followed by (35.7%) due to poor appetite and 13% due to body image concerns. This result is, however, congruent with Gajre et al. [19] and Aarthi et al. [1], who mentioned that the most common reason for skipping breakfast was lack of time, poor appetite early in the morning, and being not hungry enough to eat.

One concerning, emerging issue suggested by this study is that students are missing meals related to their efforts around body image. This is especially notable given the high rates of food insecurity reported in this sample. Growing body concerns have been noted as a globalizing trend (Brewiset al. 2018) with profound implications for how people globally relate to and decide what they eat—and hence their nutrition. This may be detecting a real phenomenon emerging in adolescents in Africa, and one that could have significant implications for how nutritional interventions are designed and implemented. That is, in a recent study with adolescents in Ibadan, Nigeria, 46.8% students who skipped breakfast similarly said they did so because they were attempting to lose weight [2].

We detected a high statistical significance between being absent from the school on other days in the prior week and skipping breakfast. More than half (56.5%) of the students who skipped breakfast that day were absent from school at least once. This result is in line with the pattern observed by kawafha in India [26] and the wider literatures connecting under nutrition to school absenteeism and academic performance of the student [37].

### Limitations of the study

There are several limitations to this study. The study depends on self-report, so there might be social desirability and recall bias from respondents, although we asked about breakfast that day to minimize the latter. Relatedly, the main variables reflect different time periods. The breakfast skipping refers to that day, whereas the data on academic performance are cumulative over a period.

The study is cross-sectional and cannot establish causation. School absenteeism may also affect the findings, in that students who are less food insecure and skipped breakfast because of that they were more likely to be absent on the date of data collection.

## Conclusion and recommendation

### Conclusion

In this case from rural Ethiopia, breakfast skipping is significantly and consistently associated with worse academic performance, even once controlling for factors like household food insecurity and income.

## Data Availability

The manuscript contains all of the data. If raw data are necessary, they will be made available upon request from the corresponding authors.
